# Usability of graphene oxide as a mycotoxin binder: In vitro study

**DOI:** 10.1371/journal.pone.0239479

**Published:** 2020-09-23

**Authors:** Pavel Horky, Eva Venusova, Tereza Aulichova, Andrea Ridoskova, Jiri Skladanka, Sylvie Skalickova

**Affiliations:** 1 Department of Animal Nutrition and Forage Production, Faculty of AgriSciences, Mendel University in Brno, Zemedelska, Brno, Czech Republic; 2 Department of Chemistry and Biochemistry, Faculty of AgriSciences, Mendel University in Brno, Zemedelska, Brno, Czech Republic; 3 CEITEC—Central European Institute of Technology, Mendel University in Brno, Zemedelska, Brno, Czech Republic; Luleå University of Technology, SWEDEN

## Abstract

Mycotoxin management in agriculture is an essential challenge for maintaining the health of both animals and humans. Choosing the right adsorbent is still a question for many breeders and an important criterion for feed manufacturers. New adsorbents are still being sought. Graphene oxide is a promising material in the field of nanotechnology, which excels in its adsorption properties. Presented in vitro study investigates graphene oxide for the binding of mycotoxins from crushed wheat. The results show that graphene oxide has an adsorption capacity for aflatoxin 0.045 mg/g, zearalenone 0.53 mg/g and deoxynivalenol 1.69 mg/g at 37° C. In vitro simulation of crushed wheat digestion showed rapid adsorption during the gastric phase. Of the minerals, Mg, Cu and Zn were the most adsorbed. The applied dose of graphene oxide of 10 mg/g caused only a slight inhibition of the digestive enzymes α-amylase and trypsin compared to pepsin and gastric lipase. In vitro results indicated the suitability of graphene oxide in the adsorption of the aflatoxin, zearalenone and deoxynivalenol.

## Introduction

Mycotoxins are a chemically broad group of compounds characterized by low molecular weight. They are usually produced by moulds, especially species Aspergillus, Penicillium, Alternaria and Fusarium. Generally, there are many species, although only a few are monitored [1]. These species pose a health risk to both humans and livestock, and their occurrence causes considerable economic damage every year. The most involved are aflatoxins (AFB1), fumonisins, ochratoxins, trichothecenes and zearalenone (ZEA) [1–3]. Their presence in the feed may occur before, after harvest, during storage and transport of crops. In general, environmental conditions such as high temperatures, high humidity, and insect damage cause stress and predispose plants in the field to mould growth and contamination of mycotoxins [4–6]. Regarding animal health, clinical signs of intoxication include gastrointestinal dysfunction, anaemia, reduction in the production parameters, reduced weight gain, lower feed efficiency and increased sensitivity to environmental and microbial stressors [7–11].

Mycotoxin adsorbents are widely used in animal production. Depending on the mode of action, they act either by binding mycotoxins to their surface (adsorption) or by degrading or transforming them into less toxic metabolites (biotransformation) using enzymes produced by bacteria, yeasts or fungi [12]. The past decade has seen the rapid development of adsorbents such as clay, activated carbon, hydrated calcium aluminosilicate and mycotoxin binding polymers. With the progress of nanotechnologies, new materials are beginning to emerge, powerful due to the large active surface [13]. These are, for example, nanomaterials based on carbon, polysaccharides, polymers or inorganic substances [14]. The adsorption efficiency depends on the chemical-physical nature of the adsorbent and chemical properties of the targeted mycotoxin. The binding efficiency of the adsorbent could be lower even by gastric juices in vivo, compared to the results obtained in the buffer in vitro. Recent investigations confirmed that the feed structure, moisture content and oxygen availability during testing might significantly affect the results of binding studies [12, 15]. However, a significant problem with mycotoxin adsorbents is undesirable adsorption of vitamins, amino acids and minerals in feed and possible risks of complexation of chemicals with mineral adsorbents [16]. Elliott et al. reported symptoms of vitamin deficiency in chickens supplemented with bentonite (0.5 to 3%) or zeolite (0.5 to 2%). Laboratory tests demonstrated reduced serum levels of zinc, copper and manganese, while aluminium concentrations increased in experimental animals [17].

In the new global economy, graphene oxide (GO) has become a central issue in several fields of the industry; energetic, optics pharmacy, medicine, sensors, contaminant removal from environment, water filtration and many others. Recent opinions predict the enormous potential of GO and advanced nanomaterials in agriculture and animal feed. GO has gained attention due to its small size, intrinsic optical properties, large specific surface area, non-toxicity, biocompatibility and useful non-covalent interactions with several organic molecules [6, 18]. Due to its excellent adsorption properties, GO can be used as an adsorbent in minimal amounts (e.g. 20–30 mg) [19, 20]. Its high popularity and the need for GO in some industries leads to the discovery of new synthesis methods that are cheap, sustainable and on a large scale. The most common Hummer method, whichburdens the environment [21, 22], is gradually being replaced by other methods such as electrochemical processes [23]. In the coming years, it can be assumed that progress in nanotechnology will also affect agriculture and GO is one of the possible options.

The principal objective of this study was to investigate the suitability of GO for the mycotoxins removal from contaminated crushed wheat. In vitro model was used for the evaluation of gastrointestinal digestion. We analyzed the adsorption efficiency of deoxynivalenol (DON), ZEA, AFB1 and also adsorption of minerals Cu, Zn, Mn, Ca, Mg and K. Finally, the research examines the impact of GO on the activity of digestive enzymes.

## Material and methods

### Chemicals

GO (size: 300–800 nm, thickness: 0.7–1.2 nm) was purchased from Cheaptubes Inc. (Cambridgeport, USA). DON, AFB1 and ZEA was purchased from MyBioSource (San Diego, USA). α-Amylase, Lipase, Bile bovine, Pancreatin, Pepsin, Trypsin-chymotrypsin inhibitor, Hemoglobin, 4-Bromophenylboronic acid, tributyrin, p-Toluene-sulfonyl-L-arginine methyl ester, and other chemicals unless noted otherwise were purchased from Sigma Aldrich (USA). 3,5-Dinitrosalicylic acid was purchased from Thermofisher Scientific (Waltham, USA). A wheat sample was obtained from the harvest of 2019. The pH value was measured using inoLab Level 3 (Wissenschaftlich-Technische Werkstatten GmbH; Weilheim, Germany). Deionised and Mili-Q water underwent demineralization by reverse osmosis using the instruments Aqua Osmotic 02 (Aqua Osmotic, Tisnov, Czech Republic). Standards of Mg, K, Ca, Mn, Zn, Cu (1g/L) were purchased from Merck (Germany). Suprapure 63% HNO_3_ and 30% H_2_O_2_ were from Sigma Aldrich (USA).

### Characterization of DON, ZEA, AFB1 binding properties to the GO

The stock solution of GO (10 mg/mL) was sonicated ten minutes in an ultrasonic bath (180 W, 40 kHz DU-45, Argolab, Carpi, Italy) until the mixture was homogenized. The stock solution of DON, ZEA and AFB1 were diluted to the final concentration 2, 12, 400 ng/g, respectively. For the absorption efficiency determination, DON was diluted in phosphate buffer saline (PBS) (pH 5) to the final concentration 15, 25, 50, 100 and 400 ng/g. ZEA was diluted in PBS (pH 6) to the final concentration 0.75, 1.5, 3, 6, 12 ng/g. AFB1 was diluted in PBS (pH 6) to the final concentration 0.25, 0.5, 1, 1.5, 2 ng/g. Subsequently, the aliquot of GO was added to reach the final concentration of 10 μg/mL. Mixtures were incubated 60 min at 37°C on the thermoblock (TS-100, Biosan, Latvia). For the time dependence adsorption (from 1 hour to 8 hours), ten μg/mL (final concentration) of GO was homogenized with DON (100 ng/g), ZEA (8 ng/g) and AFB1 (1 ng/g) at 37°C and pH 5 of PBS for DON, pH 6 of PBS for ZEA and AFB1. pH-dependent adsorption efficiency was carried out by incubating ten μg/mL of GO with DON (100 ng/g), ZEA (8 ng/g) and AFB1 (1 ng/g) at 37°C and 1 hour. PBS buffer was adjusted by HCl or NaOH to pH 2, 5, 6 and 7. Before ELISA analysis, all GO was washed from unbound mycotoxins using 3kDa Amicon cut off filters (Amicon Ultra-3K, Merck, Darmstadt, Germany). Sample filtrate was taken to the analysis.

Adsorbed amount (q) is calculated using Eq (1)

$$q\;(mg/g)=\frac{\left(c_{0}-c_{1}\right)}{m}\times V$$ (1)

% Removal was calculated by the following Eq (2):

$$\mathrm{Removal}\;(\%)=\frac{C_{0}-c_{1}}{c_{0}}\times 100$$ (2)

Where c*0* is initial mycotoxin concentration (mg/mL) and c*1* is mycotoxin concentration in the filtrate (mg/mL), V (mL) is the volume of the solution, m (g) is the mass of adsorbent used.

### Enzyme-Linked Immunosorbent Assay (ELISA)

DON, ZEA and AFB1 determination were carried out according to manufacturer description of commercial ELSA kit MyBioSource (San Diego, USA). Briefly, two grams of crushed wheat was dissolved in H_2_O for DON determination, one gram of crushed wheat was dissolved in 5 mL of 80% methanol for ZEA determination, and one gram of the crushed wheat was dissolved in 5 mL of 75% methanol for AFB1 determination. Sample supernatants were pipetted to the appropriate primary antibody-coated microtitre plate and incubated in 22°C, 15 min. Further, the wells were three times washed by washing buffer (from supplier). The secondary labelled antibodies were pipetted to the microtitre plates and incubated in 22°C, 15 min. Wells were washed three times by washing buffer. A substrate solution was added to each well, and the reaction was left for 15 min. Finally, the wells were treated by the stop solution. Mycotoxin concentration was determined by four-parameter logistic regression.

### Absorbance measurements

The samples were placed in 96 well microtitration plate (Nunc™ MicroWell™, Thermofisher Scientific, Waltham, USA). All measurements were performed at λ = 455 nm, 22° C on the Synergy HTX microplate reader (Synergy HTX, BioTek, Vermont, USA).

### Simulatory elimination of AFB1, DON and ZEN from crushed wheat

GO (10 mg/mL) was dissolved in 1 mL H_2_O and homogenized in the ultrasonic bath (180 W, 40 kHz DU-45, Argolab, Carpi, Italy). Simulated gastric fluid (SGF) and simulated intestinal fluid (SIF) were prepared according to [24]. Five g of crushed wheat (particle size 1 μm) was dissolved in 18 mL of SGF or SIF, and 2 mL of GO was added. The mixture was incubated at 37°C, 120 rpm 8 hours. The supernatant was collected and purified using 3kDa Amicon cut off filters (Amicon Ultra-3K, Merck, Darmstadt, Germany). The filtrate was collected, and unbounded DON, ZEA and AFB1 analyzed by ELISA.

### Determination of minerals by Atomic Absorption Spectrometry (AAS)

Ten μL of the wheat extract was pipetted into digestion vials. Suprapure Nitric acid (63%) and hydrogen peroxide (30%) were used as the digestion mixture. Five hundred μL of the volume of digestion mixture was used, while the ratio between nitric acid and hydrogen peroxide was always 7:3 w/w. The samples were digested by Microwave 3000 (Anton Paar GmbH, Austria), rotor MG-65. The program begins and ends with the same ten-minute-long-step and beginning with the power of 50 W and ending with the power of 0 W (cooling). Microwave power was of 100 W in the main part of the programs (for 30 min), 140°C. After the digestion, the solution of the sample was diluted to 10 mL by MilliQ water.

Cu, Zn, Mn, Ca, Mg and K were determined by 240FS Agilent Technologies atomic absorption spectrometer (Agilent, Santa Clara, USA) with flame atomization and with deuterium background correction. The instrument operated under conditions recommended by the manufacturer with air-acetylene flame (flow rate 13.5 L/min and 2.0 L/min) and using ultrasensitive hollow cathode lamp (Agilent Technologies, Santa Clara, CA, USA) as the radiation source of Zn (213.9 nm resonance line), Cu (327.4 nm), Mg (285.2 nm), Ca (422.7 nm), K (766.5 nm), and Mn (279.5 nm). The minerals concentration was determined by subtraction of the blank (without the addition of GO).

### In vitro simulation of gastrointestinal digestion

The method of in vitro simulation of gastrointestinal digestion was adopted from [24]. One g of crushed wheat was mixed with 10 mg of GO (sample) or with 10 mg of crushed wheat (blank sample). The mixture was homogenized with 5 mL of SGF at 37°C. The SGF consists of 69 mM KCl, 0.9 mM H_2_PO_4_, 25 mM NaHCO_3_, 47.2 NaCl, 0.12 MgCl_2_(H_2_O)_6_, 0.5 mM (NH_4_)_2_CO_3_, 15.6 mM HCl, 0.15 mM CaCl_2_(H_2_O)_2_, 2000 U/mL porcine pepsin and 60 U/mL gastric lipase. The mixture was incubated during moderate shaking in 37°C and 400 rpm (Orbital Shaker-Incubator, Biosan, Latvia) for 2 hours. Afterwards, 5 mL of SIF was added to the gastric chyme. The SIF consists of 6.8 mM KCl, 0.8 mM H_2_PO_4_, 85 mM NaHCO_3_, 38.4 NaCl, 0.33 MgCl_2_(H_2_O)_6_, 8.4 mM HCl, 0.6 mM CaCl_2_(H_2_O)_2_, pancreatin (100 U/mL trypsin activity) and lipase (2000 U/mL). The pH was adjusted to 7 by NaOH. The mixture was incubated 3h at 37°C and simultaneous moderate shaking 400 rpm (Orbital Shaker-Incubator, Biosan, Latvia). After incubation, the aliquot was stored at 18° C and analysed within one week by AAS and ELISA.

### Determination of enzymatic activity

The stock solution of GO (30 mg/mL) was sonicated ten minutes in ultrasonic bath (180 W, 40 kHz DU-45, Argolab, Carpi, Italy) until the mixture was homogenized. The stock solution of enzymes (1 mg/mL) was kept on the ice and mixed 1:1 with GO. As negative control, the sample without GO was prepared. The prepared samples were immediately used in the following assays.

The α-amylase activity was carried out by spectrophotometric stop reaction according to [24]. One mL of substrate solution (0.25 g/L potato starch) was incubated with 100 μL enzyme-GO solution (1 mg/mL) 3 min at 20°C. Immediately, 2 mL stop solution (96 mM 3,5-dinitrosalicylic acid dissolved 5.3 M sodium potassium tartate in 2 M NaOH) was added to the mixture and samples were incubated in 100°C to stop the reaction. Afterwards, the 9 mL of H_2_O was added, and the absorbance at λ = 540 nm was read. The results are expressed as one unit per μg of the protein releases 1 mg of maltose from starch in 3 minutes at pH 6.9 and 20°C.

Pepsine assay was adapted from [24]. Haemoglobin (0.5 g) was dissolved in 25 mL H_2_O, and the pH was adjusted to 2. 500 μL of haemoglobin to 100 μL of enzyme-GO (or blank). The mixture was incubated 10 min, and 1 mL of 5% w/v trichoroacetic acid was added. Samples were centrifuged at 6000g for 30 min. The absorbance at λ = 280 nm was read. Results were expressed as one unit per μg of the protein produced a ΔA_280_ of 0.001 per minute at pH 2.0 and 37°C.

Gastric lipase activity was adapted from [24]. Briefly, 14.5 mL of assay solution (150 mM NaCl, 2 mM sodium taurodeoxycholate, 1 μM BSA, 0.5 mL tributyrin) with enzyme-GO addition was titrated with 0.1 N NaOH. Titration was stopped at pH 5.5, and the activity of the lipase was calculated based on the rate of NaOH delivery per minute. Results were expressed as one unit per μg of the protein released 1 μmol of butyric acid per minute at 37°C and pH 5.5.

Trypsin activity assay was adapted from [24]. Three hundred μL of 10 mM p-toluene-sulfonyl-L-arginine methyl ester (TAME) was added to the assay solution (46 mM Tris/HCl buffer containing 11.5 mM CaCl_2_ at pH 8.1). One hundred μL of enzyme-GO (10 μg/mL) was added to the mixture. The absorbance at λ = 247 nm was read for 10 minutes. Results were expressed as one unit per μg of the protein hydrolysed 1 μmol of TAME per minute at 25°C and pH 8.1.

### Data treatment and descriptive statistics

The experimental work was carried out in the three independent experiments. Obtained data were presented as an average value. Results were analyzed using the ANOVA and Scheffe´s Test, and the significant result is considered at p<0.05). Data were processed using MICROSOFT EXCEL® (USA).

## Results

### Characterization of DON, ZEA, AFB1 binding properties to the GO

**Fig 1:**
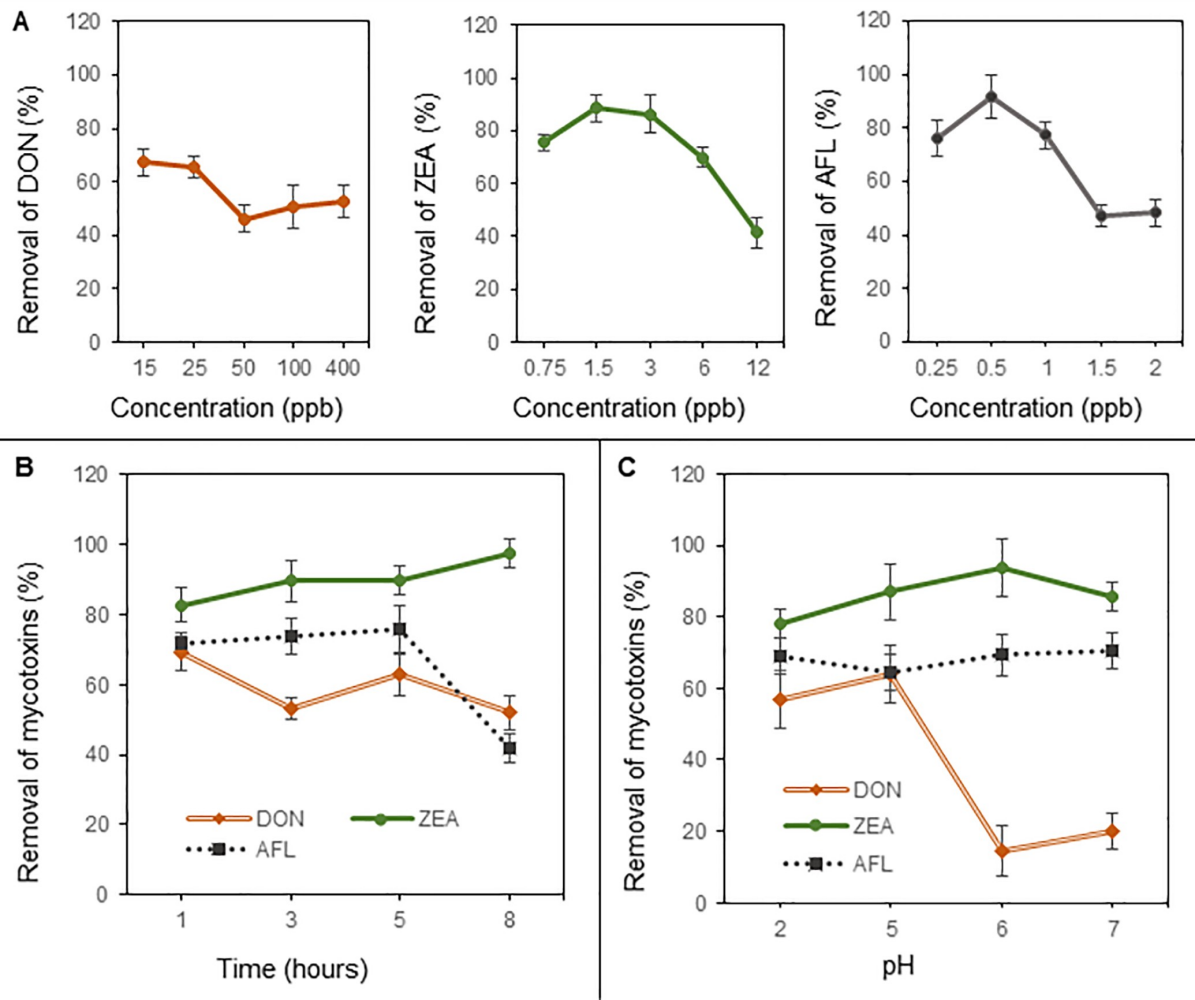
**A**) Dependence of DON (left), ZEA (middle) and AFB1 (right) concentration on removal efficiency of GO (10 μg/mL) at 37°C, 1 hour and PBS buffer pH 5 for DON, pH 6 for ZEA and AFB1. **B**) Time dependence (1–8 hours) of the removal efficiency of DON (100 ng/g), ZEA (8 ng/g) and AFB1 (1 ng/g) at 37°C and PBS buffer pH 5 for DON, pH 6 for ZEA and AFB1. **C**) Adsorption efficiency of GO in PBS buffer pH 2, 5, 6 and 7 for DON (100 ng/g), ZEA (8 ng/g) and AFB1 (1 ng/g) at 37°C and 1 hour. All experiments were performed with GO concentration of 10 μg/mL. Further details are mentioned in the materials and method section.

Most animal feed adsorbents are used by direct mixing with feed. Mycotoxin binding then occurs in the digestive tract of the animals. In our study, we first defined conditions for the removal efficiency of mycotoxins DON, ZEN, AFB1. Firstly, we monitored the removal efficiency of tested mycotoxins at the applied concentration of GO 10 μg/mL (Fig 1A). The maximal removal efficiency was attained at 65% for 25 ng/g DON, which corresponds to the adsorption capacity of 1.69 mg/g. For ZEA and AFB1 was removal efficiency around 90% for 6 and 0.5 ng/g of mycotoxins respectively. The adsorption capacity of ZEA was 0.53 mg/g and for AFB1 0.045 mg/g.

The graph on Fig 1B shows the time dependence (from 1 hour to 8 hours) of the studied mycotoxins removal by ten μg/mL GO. The mycotoxins show rapid adsorption to GO under 1 hour. ZEA and AFB1 removal slightly increases up to 5 hours of interaction. After that, there is a 10% increase in ZEA and a rapid decrease of 30% in AFB1 removal in time. In contrast, DON has shown a decrease from 65% to 50%.

GO interaction and mycotoxins were carried out in simulated gastric fluids with pH (2, 5, 6 and 7) that simulated pH across the digestive tract (Fig 1C). Whereas removal of DON showed rapid decrease up to 40% in higher pH 6 and 7, ZEA and AFB1 removal efficiency slightly increase in units of per cent.

### Simulation of gastrointestinal digestion in vitro

Firstly, the concentration of naturally occurring mycotoxins in moulded wheat was determined. The levels of DON, ZEA and AFB1 were as follows: 558.9 ng/g, 47.8 ng/g and 2.1 ng/g, respectively. Based on the calculated adsorption efficiency in the previous experiment, we mixed 10 mg GO with 1 g of crushed wheat. Subsequently, the sample was subjected to digestive process simulation. The experimental samples with GO addition were compared with the samples without GO (blank). Results were expressed as a percentage of removal.

**Fig 2:**
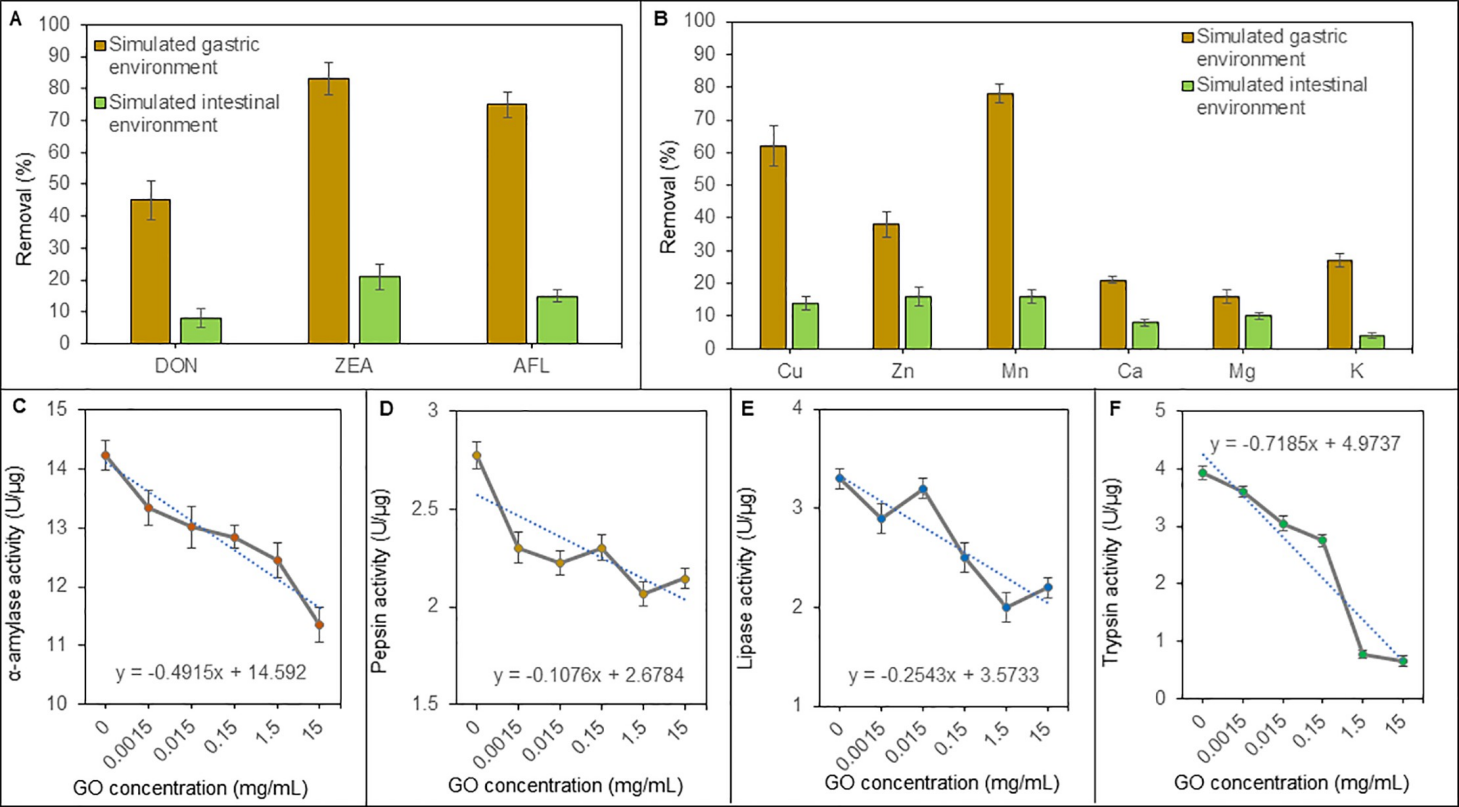
**A**) Removal efficiency of GO (10 mg/10 mL) for DON, ZEA, AFB1 from the crushed wheat (1 g). **B**) Removal efficiency of GO (1 mg/10 mL) for trace metals from crushed wheat (1 g). The incubation was carried out in 37°C 8 hours. SIF and SGF were used as an adsorption medium. The natural concentration of trace metals in the crushed wheat (100%) is as follows: Cu 0.1169 mg/kg, Zn 0.0124 mg/kg, Mn 6.37 mg/kg, Ca 0.8 g/kg, Mg 0.263 g/kg and K 0.9098 g/kg. Dependence of the GO (0–15 mg/mL) concentration on digestive enzymes activity: **C**) α-amylase, **D**) Pepsin, **E**) Lipase and F) Trypsin. The results are expressed as one unit per μg of the protein.

Fig 2A is shown the comparison of removal efficiency DON, ZEA and AFB1 in SGF and SIF. From the obtained results is evident that higher removal efficiency in SGF is at least 50% for all studied mycotoxins. The efficiency is generally lower by 30% across the pH range. GO has the lowest efficiency for DON removal and highest efficiency for ZEA and AFB1 removal.

GO is also known for its high efficiency in the binding of some metals and minerals. We carried out an analysis of essential micronutrients in samples of crushed wheat. On the graph in Fig 2B is the comparison of removal efficiency in SGF and SIF. The results show that removal is approximately 3.5 times higher in Cu, Zn and Mn removal compared to Ca^,^ Mg and K in v SGF. Cu and Mn bind the most by 60 and 80% respectively. In the simulated intestinal part of digestion, it is clear that minerals are bounded below 10%.

Furthermore, the activity of the main digestive enzymes α-amylase, pepsin, lipase and trypsin were evaluated. GO was added to the enzymes in a concentration range of 0 to 15 mg/mL. The enzymes, together with the GO, were incubated in the optimal reaction conditions. Then their activity was evaluated. The dependence of the GO concentration on the activity of the enzymes is shown in Fig 2C–2F. Data were evaluated by the regression equation. The slope of the regression equation shows the inhibition rate of the GO. Obtained results show that enzyme activity is affected, but not inhibited. A steep decline of the slope showed trypsin and α-amylase -0.7185 and -0.4915, respectively. Pepsin and gastric lipase, which act in the acidic environment of the stomach, showed a slight inhibition expressed by the slope of the regression equation: -0.1076 and -0.2543, respectively.

## Discussion

Very little was found in the literature on the question of adsorption properties of mycotoxins on GO. Several studies introduced GO as a good sensor of mycotoxins presence or for solid-phase extraction before analytical procedures. The current study found that GO has the adsorption capacity for DON, ZEA, AFB1 was 1.69, 0.53 and 0.045 mg/g, respectively. GO provided the removal effectivity was for DON 65%, ZEA and AFB1 90%. Comparison with conventional binders is difficult because their effectiveness varies. From many available studies, the adsorption capacity of GO is comparable to clays and other inorganic binders for DON, AFB1 and ZEA [12, 25–28]. From the literature is obvisous that binding atributes of GO depends on the environment. Pirouz et al. used chitosan modified GO for removal of the AFB1 B1, ZEA and ochratoxin A. Results showed the adsorption capacity of AFB1 9.6, ZEA 7.3 and ochratoxin 72.5 ng/g [29]. Although this is an order of magnitude different, adsorption was performed in a different environment. Our results are close to other work, where the adsorption capacity of magnetic GO was 5.4 mg/g for ZEA [30]. However, our work agrees that mycotoxins bind most at pH 5 (ZEA, AFB1) where is the adsorption maximum of 80–90%. Further, adsorption decreases to 60–70% at higher pH. A possible explanation of GO-mycotoxin interaction may be related to its hydrophobicity, dispersibility, solubility and polarity. The proposed adsorptive mechanism seems to be π–π interaction and hydrogen bonding between mycotoxin molecule and GO [31].

The binding properties have been evaluated in simulated gastrointestinal digestion of the crushed wheat. We evaluated it on a model that simulates the digestion of monogastric animals. The results showed an increased removal in the gastric phase. Although the removal efficiency of AFB1, ZEA and DON is lower at acidic pH, mycotoxins are bound mainly due to the incubation time. Avantaggiato et al. has investigated the dynamic of the mycotoxins removal by during gastrointestinal phases. The adsorption by various adsorbents of the tested mycotoxins (DON, ZEA and nivalenol) was more than 50% in the gastric phase. Adsorption in the ileum and jejunum was 15% lower [32]. Thus, it can be stated that the adsorption processes take place in the stomach, which is consistent with other publications [33–36]. Moreover, our results from the in vitro digestion simulation are consistent with the characterization of the binding properties of GO. Furthermore, the wheat matrix does not rapidly reduce the adsorption efficiency of mycotoxins.

A disadvantage of adsorbents is the unspecific adsorption and, hence essential nutrients might be adsorbed if their concentrations in the feed are much higher compared to those of the mycotoxin [37]. Some studies indicate that the loss of minerals can be up to 70% and must be supplemented during fattening [38–40]. GO is an excellent scavenger used in the detoxification of heavy metals, organic and inorganic pollutants. Its cross-reactivity with other feed components should be carefully considered. From our results is obvious that GO dominantly binds Mn, Ca and Zn. Electrostatic forces mediate these reactions. Strength of the interaction is given by magnitude of charges, distances between them, and on polarity of reaction environment.

The reactivity of GO with biomolecules has not been fully investigated yet. GO have been used to immobilize digestive enzymes [41–43]. However, novel findings show the GO could enhance the lipase activity in the pH dependence manner. The adsorption onto GO made the active site of the lipase accessible by altering the tertiary structure of the enzyme, leading to higher catalytic efficiency [44]. The enzymatic activity was inhibited at a concentration of 0.1 mg/mL GO in H_2_O. While GO can also be a suitable protein adsorbent, GO itself is inert to biotransformation and biological interactions [45, 46]. In our study, we verified the activity of essential digestive enzymes in the presence of GO. Their activity was partially limited but was not completely inhibited at a concentration suitable for mycotoxin binding. Our results suggest a pH-dependent interaction where the inhibition of the enzyme is lower in an acidic environment than in a higher pH environment (α-amylase and intestinal trypsin). GO is capable of interacting, depending on the environment [47]. The effectiveness of GO as an adsorbent is influenced by factors such as pH, composition and ionic strength of the environment, but also by intramolecular reactions that include steric effects. It is therefore essential to understand these phenomena in vitro and subsequently monitor them in vivo.

## Conclusion

The present study was designed to determine the efficiency of GO as a mycotoxin adsorbent. The interaction of GO and the studied mycotoxins was characterized. The findings of this study suggest that GO has an adsorption capacity for AFB1 0.045 mg/g, ZEA 0.53 mg/g and DON 1.69 mg/g at optimal pH 5 and 37°C. Adsorption capacity is not affected by the presence of a biological matrix across gastrointestinal conditions. Moreover, GO shows non-specific binding properties to micronutrients that have been adsorbed from crushed wheat by 80% Mn, 60% Cu and 40% Zn. The activity of digestive enzymes was partially limited but was not completely inhibited at a concentration suitable for mycotoxin binding. In addition to binders based on clay, activated carbon or yeast walls, GO has a promising adsorption capacity, the possibility of modification and functionalization.

## Supporting information

S1 Fig (TIF)

S2 Fig (TIF)

## References

[1] Santos Pereira C., C Cunha S., et al (2019). "Prevalent Mycotoxins in Animal Feed: Occurrence and Analytical Methods." Toxins 11(5): 290. 10.3390/toxins11050290 PMC6563184 31121952

[2] Pinotti L., Ottoboni M., et al (2016). "Mycotoxin contamination in the EU feed supply chain: A focus on cereal byproducts." Toxins 8(2): 45 10.3390/toxins8020045 26891326 PMC4773798

[3] Smith M.-C., Madec S., et al (2016). "Natural co-occurrence of mycotoxins in foods and feeds and their in vitro combined toxicological effects." Toxins 8(4): 94 10.3390/toxins8040094 27023609 PMC4848621

[4] Kolosova A. and Stroka J. (2012). "Evaluation of the effect of mycotoxin binders in animal feed on the analytical performance of standardised methods for the determination of mycotoxins in feed." Food Additives & Contaminants: Part A 29(12): 1959–1971. 10.1080/19440049.2012.720035 22971076

[5] Medina Á., Rodríguez A., et al (2015). "Climate change and mycotoxigenic fungi: impacts on mycotoxin production." Current Opinion in Food Science 5: 99–104.

[6] Abbasi Pirouz A., Abedi Karjiban R., et al (2018). "A Novel Adsorbent Magnetic Graphene Oxide Modified with Chitosan for the Simultaneous Reduction of Mycotoxins." Toxins 10(9): 361. 10.3390/toxins10090361 PMC6162667 30200553

[7] Streit E., Schatzmayr G., et al (2012). "Current Situation of Mycotoxin Contamination and Co-occurrence in Animal Feed-Focus on Europe." Toxins 4(10): 788–809. 10.3390/toxins4100788 23162698 PMC3496989

[8] Horky P., Skladanka J., et al (2016). "EFFECT OF DIET SUPPLEMENTED WITH ANTIOXIDANTS (SELENIUM, COPPER, VITAMINS E AND C) ON ANTIOXIDANT STATUS AND EJACULATE QUALITY OF BREEDING BOARS." Annals of Animal Science 16(2): 521–532.

[9] Nevrkla P., Cechova M., et al (2014). "Use of repopulation for optimizing sow reproductive performance and piglet loss." Acta Veterinaria Brno 83(4): 321–325.

[10] Magnoli A. P., Poloni V. L., et al (2019). "Impact of mycotoxin contamination in the animal feed industry." Current Opinion in Food Science 29: 99–108.

[11] Yang C., Song G., et al (2020). "Effects of mycotoxin-contaminated feed on farm animals." Journal of Hazardous Materials 389. 10.1016/j.jhazmat.2020.122087 32004836

[12] Vila-Donat P., Marin S., et al (2018). "A review of the mycotoxin adsorbing agents, with an emphasis on their multi-binding capacity, for animal feed decontamination." Food and Chemical Toxicology 114: 246–259. 10.1016/j.fct.2018.02.044 29476792

[13] Jampilek J. and Kralova K. (2020). Nanocomposites: synergistic nanotools for management of mycotoxigenic fungi.

[14] Horky P., Skalickova S., et al (2018). "Nanoparticles as a Solution for Eliminating the Risk of Mycotoxins." Nanomaterials 8(9). 10.3390/nano8090727 PMC6164963 30223519

[15] Paulick M., Rempe I., et al (2015). "Effects of Increasing Concentrations of Sodium Sulfite on Deoxynivalenol and Deoxynivalenol Sulfonate Concentrations of Maize Kernels and Maize Meal Preserved at Various Moisture Content." Toxins 7(3): 791–811. 10.3390/toxins7030791 25760079 PMC4379525

[16] Zhu Y., Hassan Y. I., et al (2016). "Innovative technologies for the mitigation of mycotoxins in animal feed and ingredients—A review of recent patents." Animal Feed Science and Technology 216: 19–29.

[17] Elliott C., Connolly L., et al (2019). "Potential adverse effects on animal health and performance caused by the addition of mineral adsorbents to feeds to reduce mycotoxin exposure." Mycotoxin Research 36: 1–12. 10.1007/s12550-019-00360-0 31515765 PMC6971152

[18] Sun X., Liu Z., et al (2008). "Nano-Graphene Oxide for Cellular Imaging and Drug Delivery." Nano research 1(3): 203–212. 10.1007/s12274-008-8021-8 20216934 PMC2834318

[19] Huang K.-J., Yu S., et al (2012). "Extraction of neurotransmitters from rat brain using graphene as a solid-phase sorbent, and their fluorescent detection by HPLC." Microchimica Acta 176(3–4): 327–335.

[20] Feizy J., Jahani M., et al (2019). "Graphene Adsorbent-Based Solid-Phase Extraction for Aflatoxins Clean-Up in Food Samples." Chromatographia 82(6): 917–926.

[21] Paulchamy B., Arthi G., et al (2015). "A simple approach to stepwise synthesis of graphene oxide nanomaterial." J Nanomed Nanotechnol 6(1): 1.

[22] Pei S., Wei Q., et al (2018). "Green synthesis of graphene oxide by seconds timescale water electrolytic oxidation." Nature Communications 9(1): 145 10.1038/s41467-017-02479-z 29321501 PMC5762692

[23] Zhang X., Zhang D., et al (2012). "Electrochemical reduction of graphene oxide films: Preparation, characterization and their electrochemical properties." Chinese science bulletin 57(23): 3045–3050.

[24] Brodkorb A., Egger L., et al (2019). "INFOGEST static in vitro simulation of gastrointestinal food digestion." Nature Protocols 14(4): 991–1014. 10.1038/s41596-018-0119-1 30886367

[25] Doll S., Danicke S., et al (2004). "In vitro studies on the evaluation of mycotoxin detoxifying agents for their efficacy on deoxynivalenol and zearalenone." Archives of Animal Nutrition 58(4): 311–324. 10.1080/00039420412331273268 15570745

[26] Di Gregorio M. C., de Neeff D. V., et al (2014). "Mineral adsorbents for prevention of mycotoxins in animal feeds." Toxin Reviews 33(3): 125–135.

[27] Kolawole O., Meneely J., et al (2019). "Comparative In Vitro Assessment of a Range of Commercial Feed Additives with Multiple Mycotoxin Binding Claims." Toxins 11(11). 10.3390/toxins11110659 PMC6891808 31726774

[28] Ji J. and Xie W. (2020). "Detoxification of Aflatoxin B1 by magnetic graphene composite adsorbents from contaminated oils." Journal of Hazardous Materials 381: 120915 10.1016/j.jhazmat.2019.120915 31352149

[29] Pirouz A. A., Karjiban R. A., et al (2018). "A Novel Adsorbent Magnetic Graphene Oxide Modified with Chitosan for the Simultaneous Reduction of Mycotoxins." Toxins 10(9). 10.3390/toxins10090361 PMC6162667 30200553

[30] Pirouz A. A., Selamat J., et al (2017). "The use of innovative and efficient nanocomposite (magnetic graphene oxide) for the reduction on of Fusarium mycotoxins in palm kernel cake." Scientific Reports 7. 10.1038/s41598-017-12341-3 PMC5622098 28963539

[31] Bai X. J., Sun C. P., et al (2018). "Detoxification of zearalenone from corn oil by adsorption of functionalized GO systems." Applied Surface Science 430: 198–207.

[32] Avantaggiato G., Solfrizzo M., et al (2005). "Recent advances on the use of adsorbent materials for detoxification of Fusarium mycotoxins." Food Additives and Contaminants 22(4): 379–388. 10.1080/02652030500058312 16019808

[33] van Rensburg C. J., Van Rensburg C. E. J., et al (2006). "In vitro and in vivo assessment of humic acid as an aflatoxin binder in broiler chickens." Poultry Science 85(9): 1576–1583. 10.1093/ps/85.9.1576 16977843

[34] Santos R. R., Vermeulen S., et al (2011). "Isotherm modeling of organic activated bentonite and humic acid polymer used as mycotoxin adsorbents." Food Additives and Contaminants Part a-Chemistry Analysis Control Exposure & Risk Assessment 28(11): 1578–1589. 10.1080/19440049.2015.1125530 21770846

[35] Gonzalez-Arias C. A., Marin S., et al (2013). "Mycotoxin bioaccessibility/absorption assessment using in vitro digestion models: a review." World Mycotoxin Journal 6(2): 167–184.

[36] Fochesato A. S., Cuello D., et al (2019). "Aflatoxin B-1 adsorption/desorption dynamics in the presence of Lactobacillus rhamnosus RC007 in a gastrointestinal tract-simulated model." Journal of Applied Microbiology 126(1): 223–229. 10.1111/jam.14101 30188600

[37] Luo Y., Liu X. J., et al (2020). "Complicated interactions between bio-adsorbents and mycotoxins during mycotoxin adsorption: Current research and future prospects." Trends in Food Science & Technology 96: 127–134.

[38] Horky P., Tmejova K., et al (2015). "Effect of Heat Stress on the Antioxidant Activity of Boar Ejaculate Revelated by Spectroscopic and Electrochemical Methods." International Journal of Electrochemical Science 10(8): 6610–6626.

[39] Nadziakiewicza M., Kehoe S., et al (2019). "Physico-Chemical Properties of Clay Minerals and Their Use as a Health Promoting Feed Additive." Animals 9(10). 10.3390/ani9100714 PMC6827059 31548509

[40] Elliott C. T., Connolly L., et al (2020). "Potential adverse effects on animal health and performance caused by the addition of mineral adsorbents to feeds to reduce mycotoxin exposure." Mycotoxin Research 36(1): 115–126. 10.1007/s12550-019-00375-7 31515765 PMC6971152

[41] Sakata M., Funatsu A., et al (2012). "Immobilization of Trypsin on Graphene Oxide Nanosheets for Increased Proteolytic Stability." Chemistry Letters 41(12): 1625–1627.

[42] Bilal M., Asgher M., et al (2019). "Multi-point enzyme immobilization, surface chemistry, and novel platforms: a paradigm shift in biocatalyst design." Critical Reviews in Biotechnology 39(2): 202–219. 10.1080/07388551.2018.1531822 30394121

[43] Zhang H., Hua S. F., et al (2020). "Effect of Graphene Oxide Prepared Under Different Conditions on Immobilized alpha-Amylase." Catalysis Letters 150(5): 1244–1255.

[44] Kalji O., Sefidbakhta Y., et al (2020). "Colloidal graphene oxide enhances the activity of a lipase and protects it from oxidative damage: Insights from physicochemical and molecular dynamics investigations." Journal of Colloid and Interface Science 567: 285–299. 10.1016/j.jcis.2020.02.010 32062491

[45] Guarnieri D., Sanchez-Moreno P., et al (2018). "Biotransformation and Biological Interaction of Graphene and Graphene Oxide during Simulated Oral Ingestion." Small 14(24). 10.1002/smll.201800227 29756263

[46] Liu S. J. (2018). "Biotransformation of graphene oxide in lung fluids significantly alters its inherent properties and bioactivities towards immune cells." Abstracts of Papers of the American Chemical Society 256.

[47] Li D. P., Zhang W. S., et al (2016). "When biomolecules meet graphene: from molecular level interactions to material design and applications." Nanoscale 8(47): 19491–19509. 10.1039/c6nr07249f 27878179

