# Supplementary figures and images for: Usability of graphene oxide as a mycotoxin binder: In vitro study
Source: PLoS One. 2020 Sep 23;15(9):e0239479. doi: 10.1371/journal.pone.0239479 (PMC7510967; doi:10.1371/journal.pone.0239479)

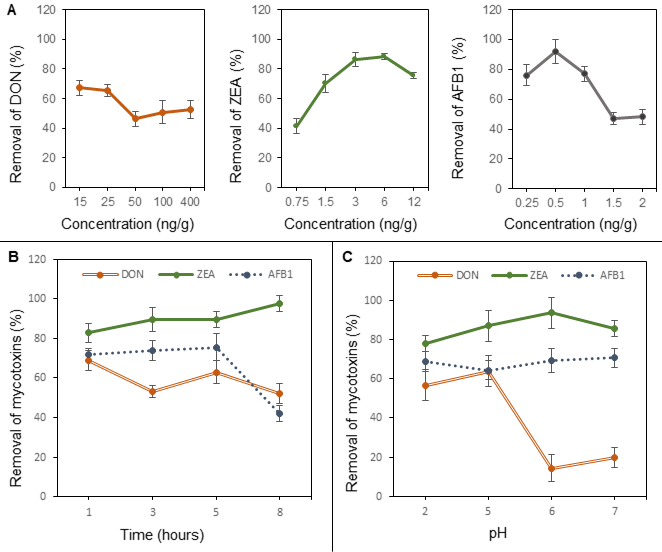

Supplement: S1 Fig — (TIF) [file pone.0239479.s001.tif]

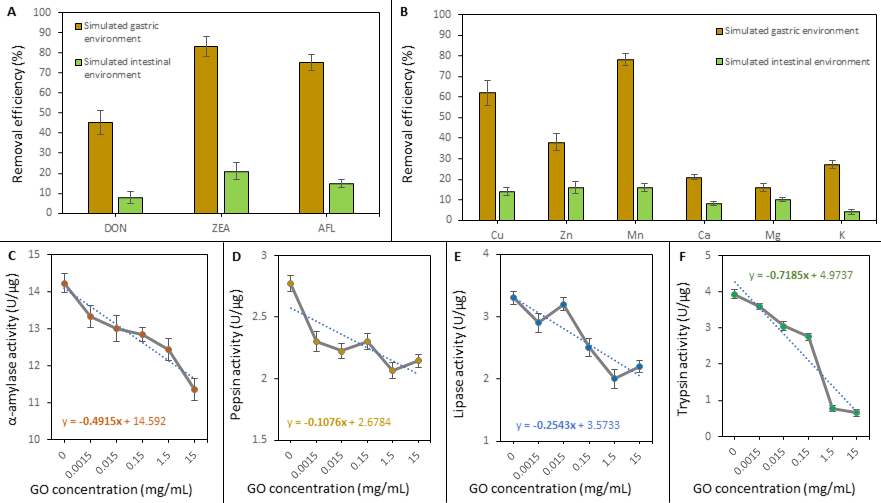

Supplement: S2 Fig — (TIF) [file pone.0239479.s002.TIF]
